# Probiotic characteristics of *Lactobacillus plantarum* E680 and its effect on Hypercholesterolemic mice

**DOI:** 10.1186/s12866-020-01922-4

**Published:** 2020-08-04

**Authors:** Zhi-yao Zheng, Fei-Wei Cao, Wei-jun Wang, Jing Yu, Chen Chen, Bo Chen, Jian-xin Liu, Jenni Firrman, John Renye, Da-xi Ren

**Affiliations:** 1 Institute of Dairy Science, College of Animal Science, Zhejiang University, Hangzhou, 310058 China; 2 Zhejiang YIMING food CO. LTD, Wenzhou, 325000 Zhejiang China; 3 Dairy and Functional Foods Research Unit, Eastern Regional Research Center, Agricultural Research Service, U.S. Department of Agriculture, Wyndmoor, PA 19038 USA

**Keywords:** *Lactobacillus plantarum* E680, Hypercholesterolemia, Cholesterol-lowering, Probiotic

## Abstract

**Background:**

Probiotics have been reported to reduce total cholesterol levels in vitro, but more evidence is needed to determine the clinical relevance of this activity. Chinese traditional fermented pickles are a good source of lactic acid bacteria. Therefore, pickle samples were collected for screening lactic acid bacteria based on their ability to survive stresses encountered during gastrointestinal passage and cholesterol reducing potency.

**Results:**

Seventy five lactic acid bacteria strains were isolated from 22 fermented pickles. From these bacteria, *Lactobacillus plantarum* E680, showed the highest acid (85.25%) and bile tolerance (80.79%). It was sensitive to five of the eight antibiotics tested, inhibited the growth of four pathogenic bacteria, and reduced the total cholesterol level by 66.84% in broth culture. In vivo testing using hypercholesterolemic mice fed high-fat emulsion, independent of food intake, found that *L. plantarum* E680 suppressed body weight gain and reduced total cholesterol and low-density lipoprotein cholesterol levels, with no effect on high-density lipoprotein cholesterol.

**Conclusions:**

Chinese traditional fermented pickles are a good source for probiotics. *L. plantarum* E680, isolated from pickles, was acid and bile tolerant, sensitive to antibiotics, and reduced cholesterol levels both in vitro and in vivo. Based on these results, *L. plantarum* E680 may have potential as a novel probiotic for the development of cholesterol-lowering functional food.

## Background

Hypercholesterolemia, or elevated serum cholesterol levels, can result from a genetic disorder affecting lipoprotein metabolism or from unhealthy lifestyle choices leading to dyslipidemia. Dyslipidemia is defined as elevated levels of low-density lipoprotein (LDL) and/or triglycerides, or low high-density lipoprotein (HDL) levels within plasma. Hypercholesterolemia, resulting from either cause is considered a major risk factor for cardiovascular disease (CVD) [1]. According to the World Health Organization (WHO), CVD is currently the world’s leading cause of death, accounting for more than 17 million fatalities each year [2]. The primary methods to reduce serum cholesterol levels, for prevention and treatment of CVD, are pharmaceuticals, dietary changes, and exercise. Statins, which inhibit hydroxy-methylglutaryl-coenzyme A reductase activity, are commonly used to reduce the levels of serum LDL-cholesterol (LDL-C). However, adverse side effects such as myotoxicity, hepatotoxicity and kidney injury have been reported due to the use of statins due to oxidative stress, and more recently, concerns have arisen in regard to their therapeutic efficacy [3]. Low-fat diets and exercise are effective approaches for lowering serum lipids and prevention of coronary atherosclerosis; however, a gap exists between targeted actual results due to consumer acceptance of low-fat diets [4]. Therefore, researchers are exploring alternative methods for lowering serum cholesterol.

Lactic acid bacteria (LAB) are ubiquitous in nature, and essential microorganisms in the production of fermentated foods, such as kimchi, dadih and cheese. In addition, several species have been investigated as probiotics for their potential to affect physiological functions within humans, including the following: immune regulation [5], alleviation of lactose intolerance [6], prevention of colon cancer and reduction of allergic reactions [7, 8].

The first report of LAB in dairy products associated with a lowering of serum cholesterol was in 1974 [9], and more researchers were attracted for its effect on preventing CVD in humans. More recently, clinical and animal studies have indicated that LAB, specifically *Lactobacillus* species, may assist in lowering serum cholesterol levels [10–12]. *L. plantarum* 299v (Pro Viva), which was isolated from the human intestinal tract, was reported to decrease viable bacteria translocation, and improve mucosal inflammation in rats [13]. In addition, this bacterium was shown to lower concentrations of LDL-C and fibrinogen in hypercholesterolemic patients [14]. *L. plantarum* LIP-1, isolated from homemade koumiss products, assimilated 71.47 μg/mL of cholesterol in vitro, and significantly reduced serum total cholesterol (TC), triacylglycerols (TG) and LDL-C levels with a concatenate increase of HDL-C in rats fed a high-fat diet [15]. In contrast to the above results, researchers have reported that some probiotic strains, which lowered cholesterol in vitro*,* did not significantly alter lipid profiles in vivo [16]. The contradicting results from previous studies supports the need for further exploration into the potential for using LAB as probiotics to lower serum cholesterol and prevent the development of metabolic diseases. Therefore, this study aimed to identify lactic acid bacteria from traditional fermented pickles, and investigate their cholesterol-lowering activity, as probiotic function in vitro. Strains displaying cholesterol-lowering activity will be further assessed for beneficial effects on hypercholesterolemic mice.

## Results

### Screening of strain with cholesterol-lowering ability

A total of 75 lactic acid bacteria isolates from 22 pickles samples were evaluated for their cholesterol-lowering capability. For the negative control (cholesterol-MRS without fermentation), the cholesterol content is 97.17 ± 2.56 μg/mL, which means the recovery percent (> 97%) of cholesterol is good for GC-MS. Five isolates were selected for further characterization due to their ability reduce cholesterol levels by more than 55% in vitro, which are similar or higher than the positive control (ATCC 43121, *Lactobacillus acidophilus*, cholesterol-reduction is 58.31%). The isolates were identified by 16S rRNA sequencing and designated as the following: *Lactobacillus plantarum* E680, *Lactobacillus fermentum* B02, *Lactobacillus plantarum* A07, *Lactobacillus brevis* H05 and *Lactobacillus acidophilus* K04 (Table 1). Of these strains, *L. plantarum* E680 displayed the highest level of cholesterol reduction (66.84%); which was significantly higher than the cholesterol-reduction observed from the control strain ATCC 43121 (*P* < 0.05).

**Table 1:** Cholesterol removal, acid resistance, and bile salt tolerance of 6 strains

Strain^1^	Cholesterol removal (%)^2^	Acid resistance (%)^3^	Bile tolerance (%)^4^
*LP* E680	66.84 ± 1.58^a^	85.25 ± 1.36^a^	80.79 ± 0.94^b^
*LF* B02	61.30 ± 3.71^b^	69.80 ± 1.65^c^	73.82 ± 2.15^c^
*LP* A07	59.81 ± 2.06^bc^	72.44 ± 1.08^c^	56.95 ± 1.79^f^
ATCC 43121	58.31 ± 3.38^bcd^	78.89 ± 0.95^b^	84.51 ± 1.05^a^
*LB* H05	56.97 ± 2.50^cd^	66.50 ± 2.09^d^	62.16 ± 2.36^e^
*LA* K04	55.21 ± 1.07^d^	80.13 ± 0.89^b^	65.12 ± 1.47^d^

^a-e^ Means in the same column followed by different superscript letters are significantly different (*P < 0.05*)

^1^Strains *LP: Lactobacillus plantarum; LF: Lactobacillus fermentum*; *LB: Lactobacillus brevis*; *LA: Lactobacillus acidophilus*

^2^Cholesterol reduction rate/% = (1-C_1_/C_0_) × 100; C_0_ is the cholesterol content in the control broth (mg/L), and C_1_ is the cholesterol content in the supernatant of the inoculated strain broth (mg/L)

^3^Acid resistance (%) = (N_1_/N_2_) × 100; N_1_ is the total number of viable cells of strain in MRS broth at 3 h, and N_2_ is the total number of viable cells of strain in MRS broth at 0 h

^4^Bile resistance (%) = (logN_1_/ log N_2_) × 100; N_1_ is the total number of viable cells of strain in MRS broth (pH 8.0) with 0.3% oxgall Bile, and N_2_ is the total number of viable cells of strain in MRS broth (pH 6.2) without oxgall Bile

### Acid and bile salts tolerance

As shown in the Table 1, acid tolerance for the five LAB isolates ranged from 66.50 to 85.25%. *L. plantarum* E680 had the highest level of acid tolerance (85.25%), while *LA* K04 displayed a similar level of tolerance to the control strain *L. acidophilus* ATCC 43121. *LB* H05 was the least tolerant (66.50%). Resistance to bile salts was also shown in Table 1, with *L. acidophilus* ATCC 43121 showing the highest level of resistance at 84.51%. *L. plantarum* E680 displayed a significantly higher level of bile salts tolerance than other strains isolated in this study at 80.79%.

### Antibacterial activity of *L. plantarum* E680

**Fig. 1:**
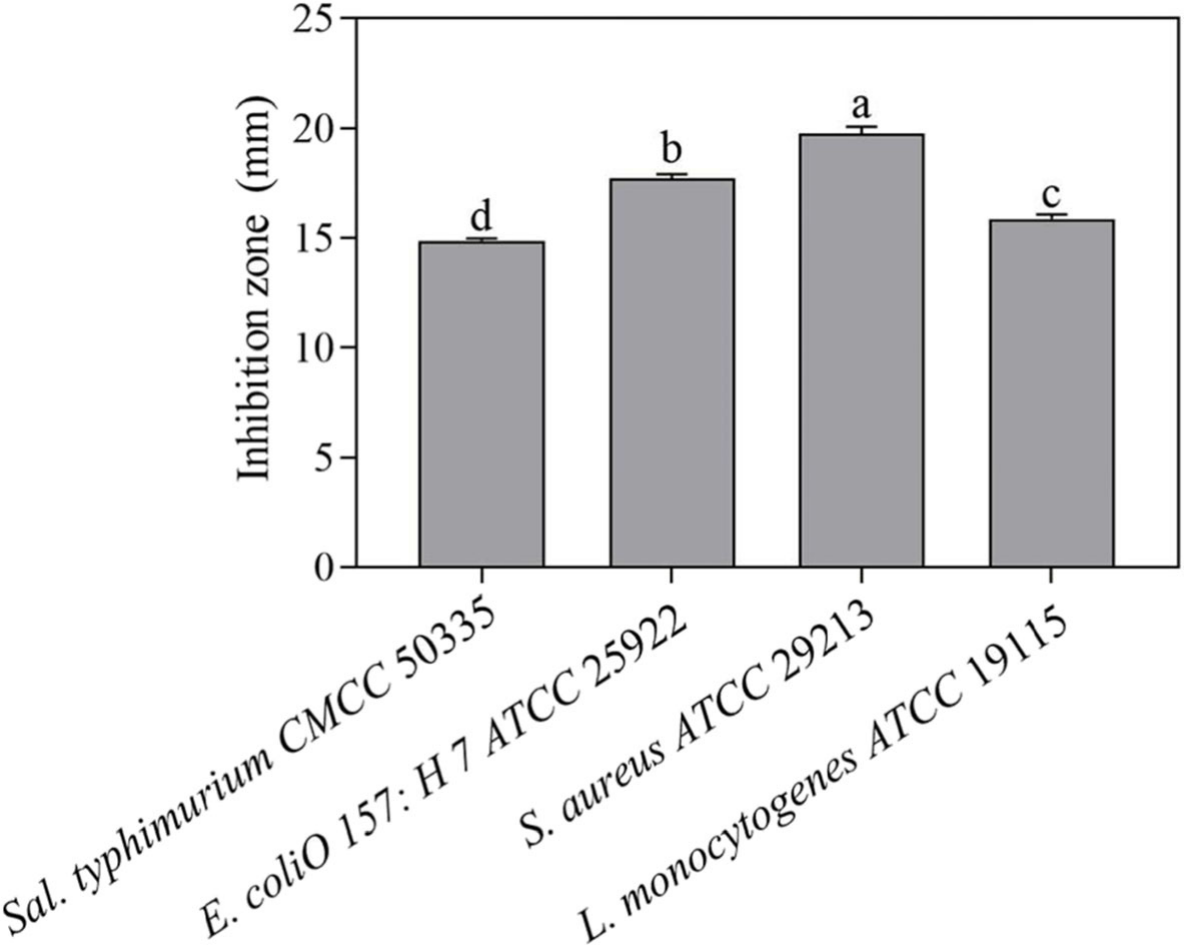
Antibacterial activity of the cell-free culture supernatant of *L. plantarum* E680 against pathogenic bacteria. Error bars represent standard deviations, different lowercase letters in each index indicate significant differences (*P <* 0.05)

*L. plantarum* E680 showed antimicrobial activity against 4 selected pathogens (Fig. 1). Activity against the four pathogens differed significantly with *Staphylococcus aureus* ATCC 29213 being most sensitive (19.8 mm inhibition zone), and *Salmonella Typhimurium* CMCC 50335 the least sensitive (14.89 mm inhibition zone). For the negative control (MRS without fermentation), no inhibition zone was found around four pathogens, which means the MRS have no effect on this four pathogens.

### Effect of *L. plantarum* E680 on mice’s body weight

**Fig. 2:**
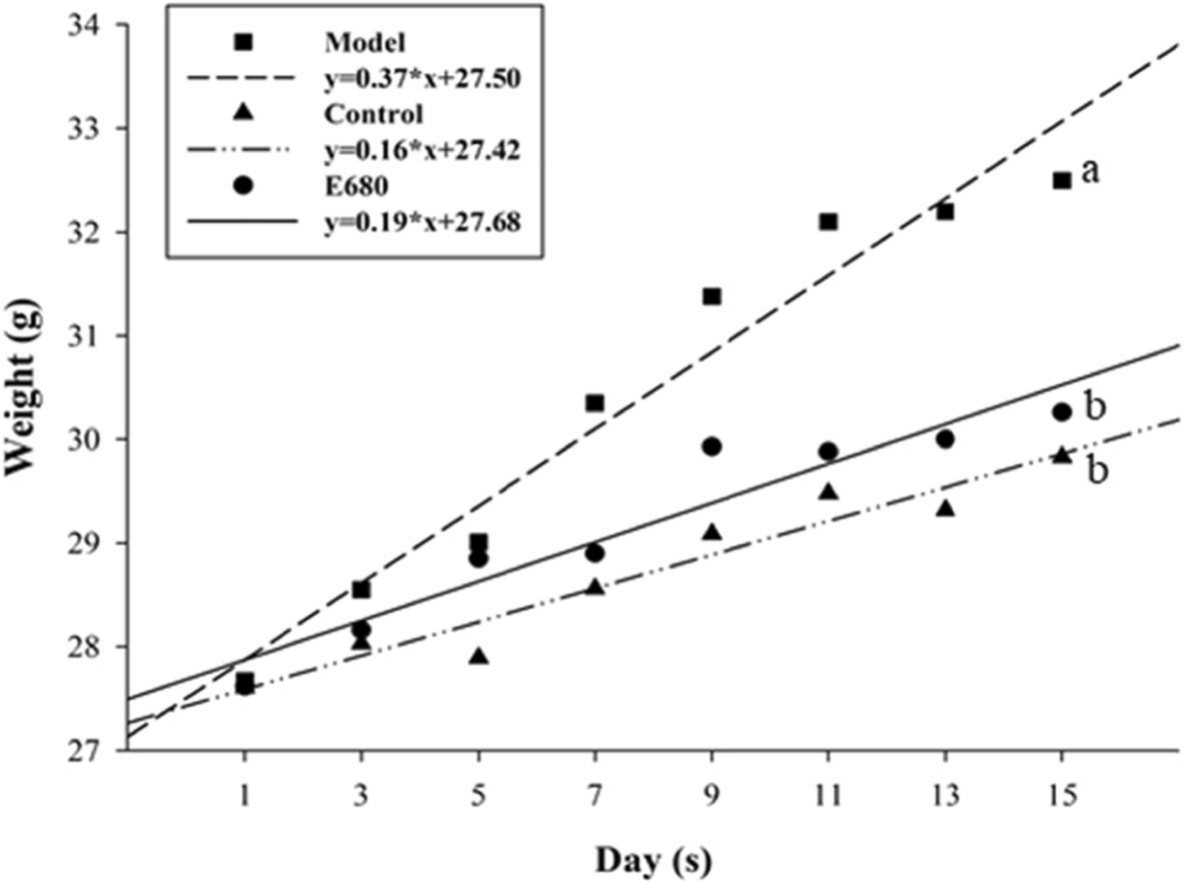
Measure of body weight (BW) within three groups of mice. Control: saline; Model: high-fat emulsion; and E680: high-fat emulsion supplemented with 10^9^ CFU/day *L. plantarum* E680. Error bars represent standard deviations with different lowercase letters indicating significant differences (*P <* 0.05, *n* = 10)

Body weight and food intake were assessed to test the potential probiotic function of *L. plantarum* E680 (Fig. 2). Initial body weight of mice was not significantly different among those receiving saline (control), a high-fat emulsion (model), or a high-fat emulsion with *L. plantarum* E680 (E680) (*P >* 0.05). However, after 2 weeks, all mice given the high-fat emulsion were significantly heavier than the control group. The body weight of *L. plantarum* E680 groups appeared similar to the control group, and significantly lower than the model group (*P <* 0.05). The slopes of linear regression equations were 0.16, 0.37 and 0.19 for the control, model, and E680 groups respectively. Differences in body weight were not due to changes in food intake levels (data not shown).

### Effect of *L. plantarum* E680 on serum lipid profiles

**Fig. 3:**
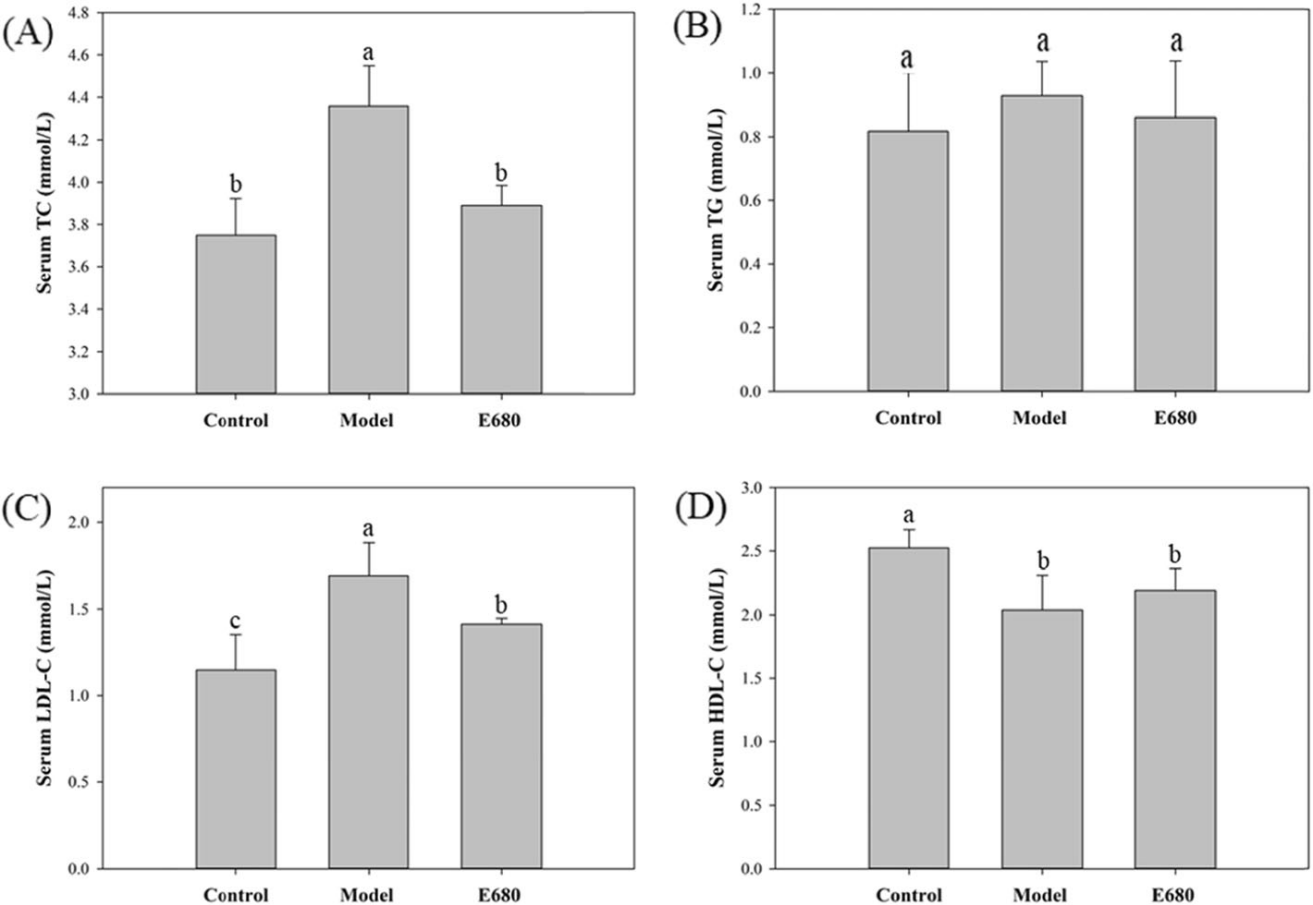
Effects of *L. plantarum* E680 on serum lipid levels of (**a**) total cholesterol (TC), (**b**) triacylglycerols (TG), (**c**) low-density lipoprotein cholesterol (LDL-C), and (D) high-density lipoprotein cholesterol (HDL-C). Error bars represent standard deviations, different lowercase letters in each index indicate significant differences (*P <* 0.05, *n* = 10)

Mice fed a high-fat emulsion diet (model) showed a significant increase in total serum cholesterol (Fig. 3a) and LDL-cholesterol (Fig. 3c) when compared to control mice receiving only saline (control). In addition, serum triglycerides levels (Fig. 3b) remained constant, and HDL-cholesterol was significantly decreased (Fig. 3d). Administering *L. plantarum* E680 with the high-fat emulsion diet prevented elevation of the total cholesterol and LDL levels, which were 10.71 and 16.47% lower than levels observed in the model group respectively (Fig. 3a and c). *L. plantarum* E680 had no effect on triglycerides levels (Fig. 3b); and was unable to prevent a drop in HDL-C levels (Fig. 3d).

## Discussion

Results of this study showed the well cholesterol-lowering activity of *L. plantarum* E680. Similar to our study, several *Lactobacillus* species, including *L. acidophilus*, *L. casei*, *L. delbrueckii* subsp. *bulgaricus*, *L. fermentum*, *L*. *gasseri*, *L. paracasei*, *L reuteri*; *L. rhamnosus*, and *L. salivarius* subsp. *salicinius*; as well as other strains of *L. plantarum*, have been reported to have cholesterol-lowering activity in vitro [17, 18]. In all of these studies, levels of cholesterol reduction were between 20% and 65%, suggesting that *L. plantarum* E680 is one of the more effective *Lactobacillus* strains possessing this activity. Although in vitro activity may identify potential probiotic candidates, one study suggested in vitro testing may not accurately reflect the cholesterol lowering activity in vivo, thus emphasizing the need for in vivo models to assess probiotic potential.

Another essential characteristic for a potential probiotic is the ability to survive passage through the gastrointestinal tract (GIT). Specifically, a probiotic must tolerate exposure to acid and bile salts for survival in the stomach and small intestine [19]. Of the strains tested in this study, *L. plantarum* E680 showed the highest tolerance to acid and bile salts. Although further studies are required to demonstrate acid and bile salt tolerance in vivo; the tolerance observed for *L. plantarum* E680 appears comparable or better than resistance reported for other *L. plantarum* strains investigated as potential probiotics. One study reported bile salts tolerance of *L. plantarum* isolates from dairy origin at 50.93 ~ 69.83% [20], while another reported acid tolerance of *L. plantarum* S04 and S10 at 73.4% and 63.2% respectively, at pH 2.5 for 3 h [21].

With bacterial antibiotic resistance being a global public health issue, there is concern about probiotics contributing to the problem due to their inherent resistance to some clinically relevant drugs [22]. In this study, *L. plantarum* E680 was tested for sensitivity to eight antibiotics, representing seven different classes (Table 2). *L. plantarum* E680 was sensitive to ampicillin (beta-lactam), cefazolin (cephalosporin), erythromycin (macrolide), sulfamethoxazole (sulfonamide), and chloramphenicol (amphenicol), with inhibition zones measuring between 16.09 and 32.41 mm in diameter; and displayed intermediate sensitivity to penicillin (beta-lactam), gentamicin (aminoglycoside) and ciprofloxacin (quinolones).

**Table 2:** Antibiotic susceptibility of *L. plantarum* E680

Class	Antibiotic	Content (μg)	Zone of inhibition diameter (mm)	Antimicrobial susceptibility type^1^
Beta-lactams	Penicillin	10	28.37 ± 0.14	I
	Ampicillin	10	29.01 ± 0.07	S
Cephalosporins	Cefazolin	30	32.41 ± 0.13	S
Aminoglycosides	Gentamicin	10	16.25 ± 0.09	I
Macrolides	Erythromycin	15	24.54 ± 0.31	S
Quinolones	Ciprofloxacin	5	16.09 ± 0.12	I
Sulfonamides	Sulfamethoxazole	1.25/23.75	22.05 ± 0.26	S
Amphenicols	Chloramphenicol	30	27.97 ± 0.15	S

^1^Inhibiton zones were compared to the breakpoints defined by CLSI (2017) [23] to determine if the bacterium was sensitive (S), resistant (R) or intermediate (I) to the antibiotics tested. Data are expressed as the mean ± SD from three replicates

Resistance to both ciprofloxacin and gentamicin has been reported in *Lactobacillus* strains used as starter cultures in Norwegian dairy products [24]; and in strains investigated as potential probiotics [25]. In these studies, resistance was considered intrinsic, with the bacteria not considered a source for the spread of antibiotic resistance to other bacterial hosts. Observations in the current study are similar to results reported for *L. plantarum* dairy isolates which were sensitive to ampicillin, chloramphenicol, erythromycin, and resistant to gentamicin [26]. However, due to the concern that LAB could serves as a potential conduit for the transfer of antibiotic resistance through food, screening for resistance to clinically relevant antibiotics is essential when screening for potential probiotics [27].

Antimicrobial activity is another potential probiotic characteristic, and *L. plantarum* E680 displayed broad spectrum activity against both Gram-positive and Gram-negative bacteria, with *S. aureus* (inhibitory zone diameter, 19.79 mm) and *E. coli* O157:H7 being the most sensitive (inhibitory zone diameter, 17.74 mm). A previous report showed *L. plantarum* Lp9 had a comparable antibacterial spectrum, however *Salmonella* Typhi and *L. monocytogenes* were more sensitive than *S. aureus* and *E. coli* [28]. Other studies have also reported that *Lactobacillus s*trains can inhibit the growth *S. aureus* and/or *E. coli* [29, 30]. The antibacterial activity of LAB may be due to organic acids (eg. lactic acid, acetic acid), hydrogen peroxide, bacteriocins, and/or exopolysaccharides produced though their metabolism [31].

In vivo analysis of the probiotic activity of *L. plantarum* E680 relied on the establishment of a hypercholesterolemic mouse model, which was successfully achieved after 14 days intragastric administration of a high-fat emulsion containing 15% cholesterol and 30% fat. The mice displayed increased total cholesterol and LDL-cholesterol levels, with normal triglycerides levels, as expected with hypercholesterolemia [1]. Previous studies have demonstrated that a high-fat emulsion diet results in lipid metabolic disorders, such as obesity, hyperlipidemia, and nonalcoholic fatty liver disease [32, 33]. This study showed that intake of *L. plantarum* E680 significantly reduced the increase of body weight caused by the high-fat emulsion in mice. Other studies reported similar results, finding that administering *Lactobacillus* to rats prevented an increase in body weight induced by a high-fat diet [34, 35]. More studies are required to determine the mechanism by which *L. plantarum* E680 prevented an increase in body weight. Other studies have shown that obesity and lipid metabolic diseases are closely related, and reported that probiotics can help prevent obesity by regulating bile salts metabolism and inhibiting dietary fat absorption in the small intestine [36, 37].

Results were comparable to those observed in previous studies, where *L. plantarum* PH04 was shown to reduce serum TC by 7% in mice [38], and the administration of *L. fermentum* M1–16 reduced TC and LDL-C levels by 12.5 and 17.3%, respectively, in rats fed a high-cholesterol diet [39]. In addition, a previous study administering probiotic blends to reduce serum TC in hypercholesterolemic rats didn’t report an increase in serum HDL-C [40], as was the case in this study. On the contrary, other studies have reported increased HDL-C levels that accompany a reduction in TC and LDL-C in hypercholesterolemic animal given probiotics [41]. More work is required to determine if the increase in HDL-C improves probiotic efficacy in preventing the development of cardiovascular disease in animal models.

Results of this study support the potential for using probiotics to control serum cholesterol levels in mice; however, more studies are required to understand the effectiveness of these bacteria in humans. One study reported that a capsule containing a probiotic blend of *L. acidophilus* and *B. longum* did not show beneficial effects on plasma lipids in young men and women with normal cholesterol levels [42]; yet a recent meta-analysis of 32 randomized controlled trials concluded that probiotic supplements could significantly reduce serum TC [43]. Several factors could explain the variations reported for the effectiveness of probiotics in controlling serum cholesterol levels, including the following: the probiotic strains being used, the host’s microbiome, and experimental protocols (probiotic dose, method of administering the probiotic, protocol for analysis of serum levels, etc.). Thus, thorough human clinical evaluations are required when trying to determine the true probiotic potential of a bacterium.

## Conclusion

In this study, 75 lactic acid bacteria isolates were found from 22 Chinese traditional fermented pickles. *L. plantarum* E680 isolated from pickles had the highest acid and bile tolerance; was sensitive to antibiotics; and was effective in reducing cholesterol levels in both in vitro and in vivo studies. Based on these results, *L. plantarum* E680 may have potential as a novel probiotic for the development of cholesterol-lowering functional food.

## Methods

### Sample collection and isolation of LAB strains

Twenty-two traditional fermented pickles were collected from farmers in Taishun, Wenzhou, Zhejiang. All samples were obtained with the farmers’ permission, and were individually packed in sterile sampling bags (Whirl-Pak® Bags, Nasco, USA) for immediate storage at 4 °C for transport to the laboratory. Ten-fold serial dilutions were prepared in sterile saline (0.85% sodium chloride), with the 10^− 5^, 10^–6,^ and 10^− 7^ dilutions (100 μL) spread onto de Man Rogosa and Sharpe (MRS) agar (Land Bridge Technology, Beijing, China). After anaerobic incubation at 37 °C for 48 h, colonies with typical morphological features were picked and subcultured on MRS agar for 3–5 passages. Isolates were further characterized by Gram-stain and catalase assays. Strains identified as lactic acid bacteria were stored in MRS broth (Land Bridge Technology, Beijing, China) with 40% (v/v) glycerol (Land Bridge Technology, Beijing, China) at − 80 °C. Prior to assays, strains were revived and passaged at least three times on sterile MRS agar.

### Identification of LAB strains

Genomic DNA was extracted with the Ezup Column Bacteria Genomic DNA Purification kit (Sangon Biotech, Shanghai, China). The 16S rDNA sequences were amplified using a bacterial universal primer set (27f: AGTTTGATCMTGGCTCAG, 1492r: GGTTACCTTGTTACGACTT) following a PCR test. The high quality PCR product obtained was sequenced by Sangon Biotech (Shanghai) Co. (Shanghai, China), and then aligned using the NCBI BLAST sequence database (http://www.ncbi.nlm.nih.gov/) to identify the species of each strain.

### In vitro cholesterol-lowering ability

LAB isolates were cultured for 18 h, harvested by centrifugation (5000 x *g*, 5 min, 4 °C) and resuspended in sterile saline solution at 10^9^ CFU/mL. The bacterial suspension was used to inoculate (2% v/v) MRS broth containing 0.3% (w/v) oxgall Bile (Sigma-Aldrich, USA) and 100 mg/L of water-soluble cholesterol (Sigmae-Aldrich, USA), and incubated anaerobically at 37 °C for 48 h. Uninoculated broth was used as a control. *Lactobacillus acidophilus* ATCC 43121 was purchased from the American Type Culture Collection (Manassas, VA, USA), and used as a positive control, due to its reported cholesterol-lowering activity [44].

Cholesterol content was determined through modification of a previously reported method [45]. Briefly, cell free supernatant was centrifuged at 5000 x *g* for 5 min at 4 °C, 1 mL was collected and passed through a 0.22 μm filter into a gas chromatography sample vial (2 mL). Samples were analyzed using an Agilent 7890A gas chromatography system (Agilent, USA) with hydrogen flame ion detector, and HP-5 elastic quartz capillary column (30 m × 0.32 mm × 0.25 μm, Agilent, USA). Split-mode (5:1), direct injection of the sample (0.5 μL) was performed using helium as the carrier gas at a flow rate of 1.5 mL/min. Initial column temperature was 100 °C and increased at a rate of 20 °C/min, reaching 300 °C at the detection port. The cholesterol-lowering activity was calculated using the formula:

$$ \mathrm{Cholesterol}\ \mathrm{lowering}\ \mathrm{rate}\ \left(\%\right)=\left(1\hbox{-} {\mathrm{C}}_1/{\mathrm{C}}_0\right)\times 100 $$

C_0_ is the cholesterol content in the control sterile broth (mg/L); C_1_ is the cholesterol content in the cell free supernatant (mg/L).

### Acid and bile salts tolerance

Acid and bile salts tolerance was determined according to the method described by previous study with minor modifications [21]. *Lactobacillus* species were cultured for 18 h, then washed twice with sterile saline, and suspended in MRS broth adjusted to pH 2.0 at a concentration of approximately 10^8^ CFU/mL. Cultures were incubated anaerobically at 37 °C for 3 h, with viable counts performed at 0 and 3 h. Separate cultures were prepared in a similar manner in MRS broth adjusted to pH 8.0 and supplemented with 0.3% (w/v) oxgall Bile (Sigmae-Aldrich, USA) at 37 °C for 6 h. Control cultures were prepared in MRS (pH 6.2) without oxgall Bile and incubated anaerobically at 37 °C for 6 h.

### Antibiotic susceptibility test

*L. plantarum* E680 was tested for antibiotic susceptibility using the disk diffusion method [46], with results analyzed according to the Clinical and Laboratory Standards Institute Technical Guidelines (2017) [23]. MRS agar was surface inoculated by evenly spreading 100 μL of bacterial culture (10^7^ CFU/mL). Disks (Table 2) (Hangzhou Microbial Reagent Co., Ltd., Hangzhou, China) were placed onto the agar surface, and plates were incubated anaerobically at 37 °C for 24 h. The diameter of the inhibition zone was measured using a vernier caliper.

### Antibacterial activity test

An agar well diffusion assay [47] was used to screen *L. plantarum* E680 for antibacterial activity against select pathogens: *Escherichia coli* O 157: H 7 ATCC 25922, *Salmonella* Typhimurium ATCC 13311 (American Type Culture Collection (ATCC), Manassas, VA, USA), *Listeria monocytogenes* CMCC 54007, and *Staphylococcus aureus* CMCC 26003 (China Medical Microorganism Culture Collection (CMCC), Guangdong, China). Molten Luria-Bertani (LB) agar (Land Bridge Technology, Beijing, China) was inoculated with a select pathogen (final concentration 10^6^ CFU/mL) and plates were poured with precast wells (8.00 ± 0.01 mm in diameter). Wells were filled with 150 μL of cell-free supernatant (18 h). Uninoculated MRS (pH 6.2) was used as control. Antibacterial activity was identified by the presence of inhibition zones.

### Animal experiments

All experiments were performed in accordance with the NIH and Zhejiang University guidelines for Laboratory Animals Care and Use and approved by the Committee for the Care and Use of Laboratory Animals at the Laboratory Animal Center, Zhejiang University (ZJU-2017-03-1).

A modified high-fat emulsion was prepared using a previously reported protocol [48]. Solid lard (30 g) was melted, and 15 g of cholesterol (Sigma-Aldrich, USA) was added, stirring constantly until fully dissolved. The solution was further supplemented with 1 g of methylthiouracil and 15 mL of Tween-80, resulting in an oil mixture. An aqueous solution was prepared by mixing 10 mL of propylene glycol and 2 g of sodium deoxycholate in 30 mL of distilled water, and heating to 60 °C. The aqueous solution was combined with the oil mixture to generate the high-fat emulsion, which was stored at 4 °C. Prior to use, the emulsion was heated to 40 °C in a water bath and homogenized.

Thirty male ICR mice, 1 month old, weighing 25 ± 2 g, were purchased from Shanghai SLAC Laboratory Animal Co., Ltd. (Shanghai, China). During the experimental period, the mice were housed in an animal room under controlled environmental conditions at a temperature of 22 ± 2 °C, relative humidity of 50 ± 5%, and a 12-h light/dark cycle, with food and water readily available. The commercial diet (Shanghai SLAC Laboratory Animal Co., Ltd., Shanghai, China) was composed of 20.5% crude protein, 4% fat, 5% crude fiber, 10% moisture, and 8% coarse ash.

**Fig. 4:**
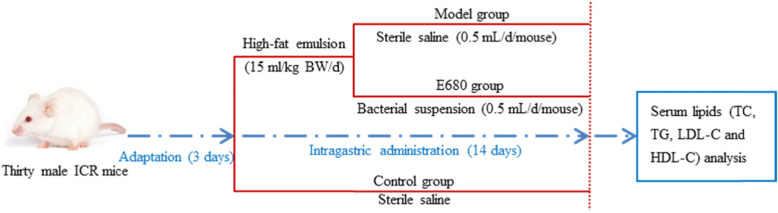
The animal experiment scheme

After 3 days of adaptation, mice were assigned to three groups (Control, Model and E680 groups) randomly, each consisting of ten mice, according to their body weight (BW). A gavage of high-fat emulsion was given to mice as previously described [49, 50]. The animal experiment scheme is illustrated in Fig. 4. Specifically, the control group was given sterile saline twice a day (0.5 mL/mouse in the morning, and 15 mL/kg BW in the evening (10 h later) by gavage. The model group received 0.5 mL of sterile saline per mouse in the morning, and intragastrical administration of 15 mL/kg BW of high-fat emulsion in the evening. The probiotic group was orally treated with a dose of 0.5 mL sterile saline containing *L. plantarum* E680 (2 × 10^9^ CFU/mL) each morning, followed by 15 mL/kg BW of high-fat emulsion in the evening. Mice were fed these diets for 14 days. Health of the mice was monitored daily, with food intake and body weight measurements recorded every 2 days.

Following 14 d of treatment, all animals were fasted for 12 h and then anaesthetized with ether (4%), the mice were sacrificed by cervical dislocation after anesthesia, and the experimental animals were not given pain during the whole process. Blood samples were collected from the orbital venous plexus into a heparinized test tube, stored at 37 °C for 1 h, and then kept at 4 °C for 30 min. Serum was obtained by centrifugation (3000 *g*, 10 min), and stored at − 80 °C for subsequent lipid analysis.

The concentration of serum lipids including total cholesterol (TC), triacylglycerols (TG), high-density lipoprotein cholesterol (HDL-C), and low-density lipoprotein cholesterol (LDL-C) were measured by commercial kits (Jiancheng, Nanjing, China) according to the manufacturer’s instructions. Ten μL of sample was mixed with working solution (37 °C, 5–10 min), then determined absorbance (510 nm) by microplate reader (Multiskan™ FC, Thermo Scientific, USA). The concentration of serum lipids was calculated based on the standard curve made from the standards.

### Statistical analysis

All experiments were repeated three times in duplicate and the results were expressed as mean ± SD. One-way ANOVA was performed on all data using SPSS professional (version 17.0). Statistical analysis was conducted using Duncan’s multiple range test (DMRT), with differences at *P < 0.05* considered statistically significant.

## Data Availability

All data generated or analyzed during this study are included in this published article.

## Abbreviations

TC: Cholesterol
TG: Triacylglycerols
HDL-C: High-density lipoprotein cholesterol
LDL-C: Low-density lipoprotein cholesterol
CVD: Cardiovascular disease
BW: Body weight
